# Toxocariasis, risk and protective factors, and mental health difficulties in early childhood: a comparison of marginalised Roma communities and the majority population

**DOI:** 10.1007/s00436-025-08557-2

**Published:** 2025-09-18

**Authors:** Daniela Fiľakovská Bobáková, Zuzana Kalinova, Elena Hatalová, Monika Halanova

**Affiliations:** 1 Department of Health Psychology and Research Methodology, Faculty of Medicine, PJ Safarik University, Kosice, Slovakia; 2 Olomouc University Social Health Institute, Palacky University in Olomouc, Olomouc, Czechia; 3 Department of Epidemiology, Faculty of Medicine, PJ Safarik University, Kosice, Slovakia

**Keywords:** Toxocariasis, Hygienic standards, Breastfeeding, Mental health, Marginalised Roma communities, Early childhood

## Abstract

Toxocariasis is a parasitic infection that poses significant health risks to children, particularly in marginalised populations with limited access to sanitation and healthcare. This study aimed to compare the occurrence of toxocariasis in early childhood between a group of children from the Slovak majority population and from marginalised Roma communities (MRCs), explore potential risk and protective factors and the association with mental health difficulties in early childhood. Cross-sectional data were obtained from mothers, and blood samples of their children aged 14–21 months were collected during the first wave of the longitudinal RomaREACH study. A total of 88 blood samples from children were analysed: 49 children from the Slovak Majority population and 39 from MRCs. Anti-*Toxocara canis* IgG antibodies were detected in serum samples using an enzyme-linked immunosorbent assay (ELISA). Though it was not statistically significant, seropositivity for *Toxocara canis* was more often observed in children from MRCs (35.9%) than in the majority population (20.4%). The absence of running water in households significantly increases the risk of *Toxocara* infection in children, whereas a longer duration of breastfeeding decreases it. Children seropositive for *Toxocara canis* showed higher levels of early mental health difficulties even when controlled for MRC residence. These results indicate the need for integrated public health interventions targeting parasitic infections in susceptible populations. Improving access to sanitation, promoting breastfeeding, and strengthening the preventive and educational role of early childhood health services are critical strategies to reduce the risk of exposure to *Toxocara* spp. eggs and mitigate its potential impact on child health and development.

## Introduction

Toxocariasis is a zoonotic parasitosis caused by *Toxocara canis* (*T. canis*) or *Toxocara cati *(*T. cati*) roundworms. Adult forms of these parasites are present in the intestines of dogs and cats, respectively. Transmission routes of *Toxocara* in a definitive host are heterogeneous. Horizontal transmission occurs through ingesting embryonated eggs or paratenic hosts. Vertical transmission occurs during pregnancy after activation of hypobiotic somatic larvae, which migrate into the developing foetus (for *T. canis*). Additionally, postpartum lactogenic transmission occurs following the migration of larvae into the mammary gland (for *T. canis* and *T. cati*) (Overgaauw 1997; Sharghi et al. 2000; Schwartz et al. 2022).

Infection in humans occurs by ingestion of embryonated *Toxocara* eggs found in contaminated environments or after the ingestion of larvae from inadequately cooked meat (Holland and Hamilton 2013). The hatched larvae penetrate the intestinal wall and are transported to the liver and lungs by the lymphatic and/or circulatory systems. From the lungs, the larvae are spread to other tissues, including the central nervous system and eyes, producing symptoms characteristic of visceral larva migrans (VLM) or ocular larva migrans (OLM) (Despommier 2003). Although the infection is often asymptomatic, clinical signs may include abdominal pain, fever, coughing, wheezing, and fatigue. Severe cases can lead to organ damage, including the liver, lungs, and brain. Infection during early childhood can impair growth and cognitive development. Severe cases may lead to neurological complications, including vision loss, seizures, and developmental delays. Chronic infections may exacerbate asthma and other respiratory conditions (Nelson et al. 1996; Hotez and Wilkins 2009; Walsh and Haseeb 2012; Inagaki et al. 2023). *T. canis* and *T. cati* do not develop into mature parasites in human hosts; therefore, eggs are not shed into the environment (Strube et al. 2013; Ma et al. 2018).

Toxocariasis is an underreported infection that is overrepresented in specific cultural groups and groups with low socioeconomic status (Mendonça et al. 2013). Several risk factors contribute to this infection, such as living in areas with poor sanitation, contact with contaminated soil or surfaces, contact with dogs and/or cats carrying *Toxocara* eggs on their fur, and consuming raw or unwashed vegetables contaminated with embryonated eggs. These risk factors are prevalent in marginalised Roma communities (MRCs). People living in MRCs are among the most disadvantaged in the EU (EU FRA 2022), facing socioeconomic deprivation, living in poor, overcrowded dwellings or housing without running water, proper sanitation, waste disposal, and veterinary infrastructure, which is limiting the ability to follow basic hygiene practices, resulting in accumulation of both animal and human faeces near homes, and facilitating soil contamination (Ihnačik et al. 2022, 2025; Pipiková et al. 2017). Several studies conducted in Slovakia have consistently demonstrated that the environment of MRCs is highly conducive to the circulation of zoonotic parasites, particularly *Toxocara* spp. Exposure to infection sources, such as stray dogs and cats, rodents, red foxes, and other wild animals, is markedly higher in these communities (Štrkolcová et al. 2017; Pipiková et al. 2017; Ihnačik et al. 2022; Antolová et al. 2004). In the most recent study, which involved over 2700 dog faecal samples and 700 soil samples from MRCs, 41.7% of dog faeces samples and 35.2% of soil samples were positive for the presence of parasite eggs. *T. canis* was among the most prevalent parasites detected (Ihnačik et al. 2025). The circulation of *Toxocara* spp. in rural Slovakia has also been documented among red foxes and small mammals, supporting the persistence of infection reservoirs beyond domestic dogs (Antolová et al. 2004).

*Toxocara* infection in early childhood can have significant health and developmental consequences if left untreated. Early detection and treatment are crucial for minimising the potential long-term effects on children’s health and development. Although some evidence of the higher prevalence of toxocariasis in MRCs exists (Antolová et al. 2015), the scope of the problem is not well documented in the period of early childhood when the basis for future health and development is laid. While the potential consequences of toxocariasis for mental health in early childhood remain largely unexplored, some studies in older children and adults have suggested possible associations of *Toxocara* infection with cognitive and mental health difficulties (Daré et al. 2019; Gale and Hedges 2020; Nelson et al. 1996; Walsh and Haseeb 2012), indicating the need for further research in younger age groups. In early childhood, mental health difficulties may manifest as problems related to excessive crying, distress, attachment, separation anxiety, aggression, or other early behavioural challenges, which can interfere with healthy development and learning and increase vulnerability later in life (Izett et al. 2021).

This study aimed to compare the occurrence of toxocariasis in early childhood between a group of children from the Slovak majority population and from marginalised Roma communities (MRCs) and explore potential risk and protective factors. Moreover, it aimed to examine the association between toxocariasis and early mental health difficulties.

## Materials and methods

The first wave of the longitudinal RomaREACH study (Research on Early Childhood in marginalised Roma communities) conducted in 2021–2022 in Prešov and Košice regions in Slovakia provided the data used in this study. These two regions were selected due to their high concentration of marginalised Roma populations, representing the areas with the largest share in Slovakia.

### Sample and procedure

Our sample consisted of 88 children aged 14–21 months. Two populations in the sample were included: 49 children from the Slovak Majority population and 39 children from MRCs. Children were recruited via paediatricians who obtained signed informed consent from the mothers and collected blood samples during mandatory preventive paediatric check-ups. Inclusion criteria required that participating mothers had sufficient cognitive capacity to complete the questionnaire and that their children were born without premature complications, birth defects, or disabilities. Mothers were asked to fill in questionnaires independently or with the assistance of researchers according to their preferences and literacy. Participation in the study was entirely voluntary and confidential.

### Measures

A series of sociodemographic variables and selected risk and protective factors were assessed. The variables included in the analyses, along with their definitions, coding, and data sources, are summarised in Table 1.

**Table 1:** Summary of measures, coding, and data sources

Measure	Type	Description	Coding/scale	Source
Residence in a marginalised Roma communities (MRCs)	Categorical (binary)	Determined based on Atlas of Roma Communities (2019), verification question on Roma neighbours, and paediatrician’s checklist	1 = MRC resident; 0 = majority population (non-MRC and not self-identifying as Roma)	Atlas of Roma Communities 2019, Office of Government Plenipotentiary for Roma Communities, Questionnaire
Running water in household	Categorical (binary)	Availability of cold running water in household	Yes/no	Gecková et al. 2014
Number of months breastfeeding	Continuous	Number of months breastfed	Months (continuous)	Questionnaire
Currently breastfed	Categorical (binary)	Current breastfeeding status	Yes/no	Questionnaire
Contact with animals	Categorical (binary)	Reported daily contact with animals (dogs, cats, etc.)	Yes/no	Questionnaire
Contact with un-dewormed animals	Categorical (binary)	Regular contact with animals not regularly checked by a veterinarian (dewormed and vaccinated)	Yes/no	Questionnaire
Early mental health difficulties	Continuous (score)	Mental Health subscale of CREDI for children < 3 years; measures aggression, anxiety, distress	Sum score 0–9; higher = more frequent symptoms	McCoy et al. 2018a, 2018b; McCoy et al. 2017
*Toxocara canis* seropositivity	Categorical (binary)	Detection of anti-*Toxocara canis* IgG antibodies in serum via ELISA	Negative: < 9 U; equivocal: 9–11 U; positive: > 11 U	Demeditec Diagnostics GmbH, Germany

Residence in a marginalised Roma community as a determinant variable was assessed based on the Atlas of Roma Communities—the sociographic Mapping conducted by the Office of the Government Plenipotentiary for the Roma Communities in cooperation with the Institute for Work and Family Research undertaken by the Ministry of the Interior of the Slovak Republic in 2019 as well as by the verification of the question ‘Are your closest neighbours mostly Roma?’ Additionally, paediatricians who collected blood samples indicated the residence of children in MRCs on a brief checklist. The majority population refers to inhabitants outside of marginalised Roma communities who, at the same time, do not consider themselves Roma.

The availability of amenities in the household (Gecková et al. 2014) was asked using the question: ‘Is the following in your household? (cold running water, hot running water, working flushing toilet, working bathroom or shower, and electricity)’. The response categories were ‘yes’ and ‘no’. The item covering running water was used for the present study.

Breastfeeding was assessed by asking about the number of months of breastfeeding and whether the child is still breastfed (yes/no).

To assess the risk resulting from contact with animals, two separate questions were used: (1) ‘Do you meet animals (dogs, cats, etc.) daily? (yes/no)’ and (2) ‘Do you regularly come into contact with animals (cats, dogs, etc.) that are not regularly checked by a veterinarian (dewormed and vaccinated)? (yes/no)’.

Early mental health difficulties were assessed using the Mental Health subscale of the Caregiver Reported Early Development Instrument (CREDI), which was designed to measure development in children under 3 years of age (McCoy et al. 2018b) and to be used in low-resource settings (McCoy et al. 2017). The mental health subscale captures early symptoms of children’s mental health difficulties, including behaviours related to aggression, anxiety, and distress (McCoy et al. 2018a). The sum score ranged from 0 to 9 (Cronbach’s *α* = 0.64). A higher score indicates more frequent emotional and behavioural symptoms.

### Blood sample collection and analysis

Venous blood samples from the children were obtained following standard procedures, collected into 4.4 ml tubes containing a clot activator and separation gel, and immediately transported to the laboratory for serum extraction. The serum was subsequently isolated by centrifuging the blood samples at 3000 rpm for 15 min using an iFUGE D06 centrifuge (Neuation). Collected sera were stored at −20 °C until analysis. For the determination of anti-*Toxocara canis* antibodies in serum samples, the *Toxocara canis* IgG Elisa kit (Demeditec Diagnostics GmbH, Germany) was used according to the manufacturer’s instructions. The optical density (OD) was measured using the BioTek ELx800 Absorbance Microplate Reader (BioTek® Instruments, Inc., USA) at a wavelength of 450 nm as well as a reference wavelength of 620 nm to exclude background signals. Results in Units (U) were calculated and interpreted as follows: negative result: < 9 U, equivocal result: ≥ 9 U to ≤ 11 U, positive result: > 11 U.

### Statistical analyses

First, the demographic and socioeconomic characteristics of the sample and the risk and protective factors were characterised using descriptive statistics. Differences between MRCs and the majority, as well as between seronegative and seropositive children, were assessed using the Chi-squared test for dichotomous variables and Mann–Whitney U-test for continuous variables, as the distribution of these variables was not normal according to Kolmogorov–Smirnov and Shapiro–Wilk tests.

Next, we assessed the association between risk and protective factors and seropositivity for *T. canis* infection in children using simple logistic regression. Finally, the association of *T. canis* infection seropositivity in children with early mental health difficulties (Model 1) was explored and adjusted for MRC residence (Model 2) using linear regression. A *p*-value lower than 0.05 was considered significant. Statistical analyses were performed using IBM SPSS 23 for Windows.

## Results

Sociodemographic, environmental, and health-related characteristics of the sample can be found in Table 2. Significant differences were found between children from the majority population and MRCs in several variables reported by mothers. These included lower contact with un-dewormed animals, better access to running water, a higher likelihood of being currently breastfed, longer duration of breastfeeding, and fewer early mental health difficulties among children in the majority population compared with those from MRCs. Regarding *T. canis* seroprevalence within each population group, 35.9% of children from MRCs tested seropositive, compared to 20.4% of children from the majority population. The difference in prevalence did not reach statistical significance.

**Table 2:** Sociodemographic, environmental, and health-related characteristics of children from MRCs and the majority population

	**Majority** *N = *64		**MRCs** *N* = 24		**Total** *N* = 88		***p*-value**
	***N***	**(%)**	***N***	**(%)**	***N***	**(%)**	
Child’s sex							0.716
Boys	22	44.9	16	41.0	38	43.2	
Girls	27	55.1	23	59.0	50	56.8	
Contact with animals	24	50.0	26	68.4	50	58.1	0.085
Contact with un-dewormed animals	2	4.3	15	41.7	17	20.7	0.000
No running water	1	2.1	15	39.5	16	18.6	0.000
Currently breastfed	20	41.7	7	18.4	27	31.4	0.021
*Toxocara canis* seropositive	10	20.4	14	35.9	24	27.3	0.105
	Median (IRQ)		Median (IRQ)		Median (IRQ)		
Age of children in months	16.00 (2.00)		16.00 (2.00)		16.00 (2.00)		0.889
Number of months breastfeeding	12.00 (13.00)		2.00 (13.00)		6.00 (14.00)		0.000
Early mental health difficulties	2.00 (2.00)		4.00 (2.00)		3.00 (2.00)		0.000

Statistically significant differences between *T. canis* seropositive and seronegative children (Table 3) can be seen in the number of months breastfeeding and current breastfeeding, absence of running water in the household, and early mental health difficulties. Among the children who were seropositive for *Toxocara canis* antibodies, 58.3% belonged to MRCs, while 41.7% were from the majority population.

**Table 3:** Comparison of sociodemographic, environmental, and health-related characteristics between *Toxocara canis* seropositive and seronegative children

	***Toxocara***						
	**Seronegative** *N* = 64		**Seropositive** *N* = 24		**Total** *N* = 88		***p*-value**
	***N***	**(%)**	***N***	**(%)**	***N***	**(%)**	
Child’s sex							0.758
Boys	27	42.2	11	45.8	38	43.2	
Girls	37	57.8	13	54.2	50	56.8	
MRC residence	25	39.1	14	58.3	39	44.3	0.105
Contact with animals	35	56.5	15	62.5	50	58.1	0.610
Contact with un-dewormed animals	12	20.0	5	22.7	17	20.7	0.787
No running water	8	12.9	8	33.3	16	18.6	0.029
Currently breastfed	24	38.7	3	12.5	27	31.4	0.019
	Median (IRQ)		Median (IRQ)		Median (IRQ)		
Age of children in months	16.00 (1.00)		16.00 (2.00)		16.00 (2.00)		0.569
Number of months breastfeeding	11.00 (14.00)		2.50 (12.00)		6.00 (14.00)		0.033
Early mental health difficulties	2.00 (3.00)		4.00 (3.00)		3.00 (2.00)		0.008

### Association between risk and protective factors and toxocariasis infection among children

Logistic regression shows that each additional month of breastfeeding reduces the odds of testing positive for *T. canis* infection (OR = 0.92; 95% CI: 0.85–0.99). Similarly, current breastfeeding was associated with lower odds of *T. canis* seropositivity (OR = 0.23; 95% CI: 0.06–0.84). Lack of running water was associated with higher odds of *T. canis* seropositivity (Table 4).

**Table 4:** Logistic regression analysis: association of risk and protective factors with *Toxocara canis* seropositivity in children

	**Crude effect** OR (CI)
No running water	3.38 (1.09–10.42)*
Currently breastfed	0.23 (0.06–0.84)*
Number of months breastfeeding	0.92 (0.85–0.99)*
Age of children in months	0.88 (0.58–1.35)

* *p < *0.05

### Association between toxocariasis and early mental health difficulties

As shown in Table 5, children tested seropositive for *T. canis* showed significantly higher levels of early mental health difficulties manifesting as more frequent emotional and behavioural symptoms (*B* = 1.40; 95% CI: 0.43–2.37), even when controlled for MRC residence (*B* = 0.99; 95% CI: 0.05–1.94) or current breastfeeding (*B* = 1.24; 95% CI: 0.24–2.24). Additionally, MRC residence was independently associated with more early mental health difficulties (*B* = 1.37; 95% CI: 0.50–2.24).

**Table 5:** Linear regression analysis: association of *Toxocara canis* seropositivity with early mental health difficulties (Model 1) and adjusted for MRC residence (Model 2) and current breastfeeding (Model 3)

	**Model 1—Crude effect** *B *(CI)	**Model 2** *B* (CI)	**Model 3** *B* (CI)
*Toxocara canis* seropositivity	1.40 (0.43–2.37)**	0.99 (0.05–1.94)*	1.24 (0.24–2.24)*
MRC residence		1.37 (0.50–2.24)**	
Currently breastfed			−0.62 (−1.60–0.35)

* *p < *0.05, ** *p* < 0.01

In the post-hoc analyses, we tested whether current breastfeeding moderates the association between *T. canis* seropositivity and early mental health difficulties. The interaction showed a negative coefficient and a *p*-value at the borderline of statistical significance (*B *= –2.264; *p* = 0.071).

## Discussion

The present study compared the occurrence of *T. canis* infection between children from the Slovak majority population and those from MRCs. Additionally, it assessed the effects of selected risk and protective factors on the occurrence of *T. canis* infection in children and explored its potential impact on early mental health difficulties. Although the prevalence of *T. canis* seropositivity was higher among children from MRCs than children from the majority population, this difference did not reach statistical significance. This finding suggests that, while children from MRCs may experience increased environmental exposure to risk factors, *T. canis* infection is not limited to these populations. It highlights that parasitic diseases such as toxocariasis can affect children across different social and ethnic groups, particularly in settings where access to sanitation and environmental hygiene, including cleaning up after pet defecation, is inadequate. This underscores the need for universal prevention strategies, including proper deworming of pets, improved public sanitation, and adherence to hygiene regulations. In Slovakia, Decree No. 521/2007 Coll. (Ministry of Health of the Slovak Republic 2007) sets hygiene and maintenance standards for public spaces, including sandpits, which are known reservoirs of *T. canis* eggs if not adequately maintained. Strengthening the implementation of such regulations could contribute to reducing environmental contamination and infection risk among all children, regardless of socioeconomic background. The lack of statistical significance may also be attributed to the sample size or unmeasured confounding factors. Nevertheless, these results emphasise the need for universal prevention strategies to reduce parasitic infections in all susceptible populations rather than focusing solely on marginalised groups.

Our findings demonstrate a significant association between *T. canis* seropositivity and several socioeconomic and environmental risk factors in children. Lack of basic sanitary infrastructure—precisely the absence of running water—was identified as a key determinant of infection. These results are consistent with existing evidence that poor environmental hygiene facilitates the transmission of soil-transmitted helminths through poor living conditions with exposed dirt floors, contact with contaminated soil, and exposure to stray domestic animals without proper veterinary control and roaming freely all over the MRCs and their surroundings (Palmieri et al. 2018; Jarošová et al. 2021; Ihnačik et al. 2025). This is supported by findings from Slovakia, where Antolová et al. (2015) reported a significantly higher *Toxocara* seroprevalence in Roma adults (22.1%) compared to the non-Roma population (1.0%) and identified inadequate access to water, sanitation, and hygiene as a Major risk factor. They found no association between age and seropositivity in the non-Roma population, while in the Roma population, seroprevalence increased with age, possibly reflecting cumulative exposure and repeated infections. This suggests that individuals in MRCs experience continuous environmental infectious pressure, which May contribute to persistently elevated antibody levels. Given that the children in our study were 14–21 months old, it is unlikely that the observed *Toxocara canis*-specific IgG seropositivity reflects maternally derived antibodies. Transplacental antibodies typically reach low or undetectable levels within the first year of life, and breast milk contains mainly secretory IgA (Gibbs and Fairfax 2022), supporting the interpretation that seropositivity reflects postnatal exposure to *Toxocara* spp. in early childhood. The absence of clean water increases the likelihood of exposure to infectious agents, particularly in young children who are more likely to play in contaminated environments. Even when sanitary facilities are available, young children remain highly susceptible due to underdeveloped hygiene habits, frequent hand-to-mouth behaviour, and geophagia, which increase the risk of infection (Glickman et al. 1999; Morita et al. 2017). Addressing these deficits is, therefore, essential. Public health strategies prioritising access to clean water, improved sanitation, and hygiene education are critical to reducing the risk of parasitic infections in susceptible populations (Brown et al. 2023).

Another factor found to be associated with *T. canis* seropositivity was the shorter duration of breastfeeding. Breastfeeding is widely recognised for its immunological and nutritional benefits, including protective effects against various intestinal parasitic infections (Ali et al. 2024). The lower prevalence of intestinal parasitic infections among breastfed (particularly exclusively breastfed) children has been attributed not only to reduced exposure to contaminated food and water, but also to the immunologically active components of breast milk. These include secretory IgA, lactoferrin, lysozyme, and growth factors, which contribute to maintaining gut mucosal integrity and limiting pathogen adherence and invasion (Kutty 2014). While most evidence focuses on protozoan and some helminth infections, these mechanisms may plausibly extend to zoonotic helminths such as *Toxocara* spp., although direct protective effects against *Toxocara* have yet to be established. Given the high seroprevalence in adult Roma women (Antolová et al. 2015), it is biologically plausible that maternal antibodies may be transferred to infants, potentially providing limited passive protection during early infancy. However, this remains hypothetical and warrants confirmation in future studies. Nevertheless, promoting breastfeeding as a culturally acceptable and accessible protective practice may therefore not only improve general child health but also further decrease exposure risk and strengthen early immune defences (Ferreira et al. 2007; Kutty 2014; Ali et al. 2024).

Importantly, *T. canis* seropositivity was also associated with increased early mental health difficulties in children. Disadvantaged children were previously shown to exhibit more symptoms of impaired mental health (Filakovska Bobakova et al. 2024) and are also breastfed for significantly shorter periods (Fiľakovská Bobáková and Dankulincová Veselská 2024). Even after adjusting for residence in MRCs and current breastfeeding, children who tested positive for *Toxocara canis* antibodies exhibited higher levels of early mental health difficulties. While adjusting for residence in MRCs, this may not fully account for the multiple disadvantages these children face, and the findings should therefore be interpreted within this broader context of structural and social determinants of health. Prior research highlights that children from socioeconomically deprived environments are exposed to cumulative risks such as poverty, maternal stress, substandard housing, and limited access to healthcare (e.g. Evans et al. 2004; Shonkoff et al. 2021; Fiľakovská Bobáková et al. 2024). These co-occurring adversities may confound or amplify the association between biological exposures such as *T. canis* infection and child mental health outcomes. Although the exact mechanisms remain to be fully understood, chronic parasitic infections are hypothesised to contribute to neurodevelopmental disturbances through pathways such as persistent immune activation, neuroinflammation, and potential direct effects on the central nervous system. These processes may increase the risk of emotional and behavioural problems, cognitive impairments, and delays in developmental milestones (Alloo et al. 2022; de Brito Duval et al. 2025).

The role of breastfeeding in early childhood mental health is multifaceted, encompassing not only nutritional and immunological benefits but also psychosocial influences such as maternal bonding and stress regulation (Modak et al. 2023). In our post-hoc analyses, a potential interaction was observed suggesting that breastfeeding may attenuate the adverse effects of *T. canis* infection on mental health. Although this trend did not reach statistical significance, it was close to the conventional threshold (*p = *0.071), warranting further investigation. This highlights the need for additional research into the possible protective role of breastfeeding, particularly in vulnerable populations. Overall, our findings underscore the need to recognise parasitic infections not only as infectious diseases but also as risk factors for broader developmental vulnerabilities. Integrated public health approaches that combine parasite control with early childhood mental health screening and support services are essential to mitigate these long-term impacts. Drawing on evidence from both human studies and animal models, future research should explore the causal pathways between *T. canis* infection and child neurodevelopment. Studies in older children and adults have reported associations between *Toxocara* infection and cognitive as well as mental health difficulties (Daré et al. 2019; Gale and Hedges 2020; Nelson et al. 1996; Walsh and Haseeb 2012). Experimental research in mice has shown that brain infection with *T. canis* can alter exploratory behaviour, increase anxiety, and impair learning (Cox and Holland 2001), supporting the biological plausibility of such effects in humans. Evaluating the effectiveness of comprehensive interventions in reducing these adverse outcomes should remain an important priority.

### Strengths and limitations

A significant strength of this study is its focus on a high-risk population with limited access to healthcare and an increased burden of infectious diseases. Including very young children—aged 14–21 months—in serological testing for *T. canis* infection is particularly noteworthy, as such studies are rare in this age group. Furthermore, the successful inclusion of participants from MRCs, who are frequently underrepresented in health research, enhances the relevance of our findings for addressing health inequities in susceptible populations. Additionally, the study identifies an association between breastfeeding and a lower prevalence of *T. canis* infection, which may partly reflect other underlying protective factors and remains underexplored in the existing literature. By examining both socioeconomic and environmental risk factors and early mental health outcomes, this study offers a more comprehensive understanding of the multifaceted impact of *T. canis* infection in early childhood.

However, several limitations must be acknowledged. The cross-sectional design precludes causal inferences, and the reliance on IgG seropositivity as an exposure indicator does not allow differentiation between recent and past infections. Furthermore, data on hygiene practices, environmental contamination (such as soil or animal faeces testing, visitation of sylvatic carnivores), or deworming practices of household pets were not collected. These could have provided further clarity regarding the transmission pathways. In addition, potential reporting bias related to self-reported measures of contact with un-dewormed animals should be considered when interpreting the results. Although contact with un-dewormed animals was considered a possible risk factor, a statistically significant association was not found. The lack of association with this environmental factor may raise questions about the dominant transmission routes. While human *Toxocara* infection is primarily associated with exposure to contaminated soil, transmission via the fur and paws of animals is a topic of debate (Holland 2017; García-Rubio et al. 2023). Although stray dogs show a higher prevalence of *Toxocara* eggs on fur (Holland 2017; Salawu and Akeredolu 2023), studies also demonstrate that owned and dewormed dogs can acquire eggs from contaminated environments and passively transport them indoors (Maurelli et al. 2019; Panova and Khrustalev 2018). However, the proportion of embryonated, infective eggs on fur is generally low (Holland 2017; Maurelli et al. 2019). Nevertheless, the lack of association May also be attributed to the limited sample size of 88 children from whom blood samples were collected, which reduces the statistical power to detect significant associations, particularly in subgroup analyses. Additionally, the interaction between *T. canis* seropositivity and current breastfeeding did not reach statistical significance, potentially due to the limited sample size. As the result was near the conventional threshold, it may indicate an actual moderating effect that should be explored in larger samples. This sample size also limits the generalizability of the findings beyond the study population. Lastly, the internal consistency of the Mental Health subscale of the CREDI was modest (Cronbach’s *α* = 0.64), which may reflect the challenge of reliably capturing a broad range of early emotional and behavioural symptoms in very young children through caregiver reports. This should be taken into account when interpreting associations involving this outcome.

### Implication

The findings of this study have important implications for public health policies and interventions aimed at reducing the burden of parasitic infections among susceptible populations. The association between *T. canis* seropositivity and inadequate sanitation highlights the critical need to improve basic living conditions in MRCs, particularly access to clean water. Addressing this infrastructural deficit should be a priority to mitigate environmental risk factors contributing to parasitic infections. Moreover, deworming animals at high risk of environmental exposure (due to free roaming, close contact with other animals, hunting behaviour, or coprophagy), as is often the case in MRCs, should be extended from at least four times a year to up to monthly treatment (or undergo faecal examinations at the same frequency followed by targeted treatment if positive) to interrupt the parasite’s life cycle, prevent egg shedding, and effectively reduce the risk of *Toxocara* infection (ESCCAP 2025).

Furthermore, the observed association between breastfeeding and reduced seropositivity suggests that health promotion strategies could consider the potential protective role of prolonged and exclusive breastfeeding, particularly in high-risk populations. While the underlying mechanisms in relation to *Toxocara* infection remain unclear and may be indirect, interventions that support breastfeeding through education and resources could form part of broader child health promotion strategies in these settings.

The observed association between *T. canis* seropositivity and early mental health difficulties highlights a potential link worth further investigation, given the broader developmental and neuropsychological impacts reported for some parasitic diseases. Public health programs in MRCs could integrate parasite control efforts with child development and mental health services to address overlapping vulnerabilities. Future longitudinal research is needed to clarify the direction and nature of these associations and to inform interventions targeting both environmental and social determinants of health in MRCs.

## Conclusion

Environmental deprivation (poor sanitation, lack of clean water) and limited breastfeeding are associated with higher *T. canis* seropositivity. *T. canis* seropositivity is further associated with early mental health difficulties, raising concerns about the broader developmental impact of parasitic disease. These results indicate the urgent need for integrated public health interventions targeting parasitic infections in susceptible populations. Improving access to sanitation, promoting breastfeeding, implementing regular deworming of dogs, and ensuring early childhood health services are critical strategies that may help reduce the burden of *T. canis* infection and its potential impact on child health and development. Attention should also be given to enforcing hygiene standards, as inadequate adherence remains a concern, especially in environments such as playgrounds, where children are highly exposed. Strengthening the implementation and monitoring of these standards could help minimise environmental contamination and reduce infection risks.

## Data Availability

The data supporting this study is available from the corresponding author upon request.
